# The trade-off between heat tolerance and metabolic cost drives the bimodal life strategy at the air-water interface

**DOI:** 10.1038/srep19158

**Published:** 2016-01-13

**Authors:** Marco Fusi, Stefano Cannicci, Daniele Daffonchio, Bruce Mostert, Hans-Otto Pörtner, Folco Giomi

**Affiliations:** 1 King Abdullah University of Science and Technology (KAUST), Red Sea Research Center, Thuwal, 23955-6900, Saudi Arabia; 2 Department of Biology, University of Florence, Sesto Fiorentino, Italy; 3 The Swire Institute of Marine Science and the School of Biological Sciences, the University of Hong Kong, Pokfulam Road, Hong Kong; 4 Department of Zoology and Entomology, Rhodes University, Grahamstown, Africa; 5 Department Integrative Ecophysiology, Alfred-Wegener-Institute for Polar and Marine Research, Bremerhaven, Germany

## Abstract

The principle of oxygen and capacity limitation of thermal tolerance in ectotherms suggests that the long-term upper limits of an organism's thermal niche are equivalent to the upper limits of the organism's functional capacity for oxygen provision to tissues. Air-breathing ectotherms show wider thermal tolerances, since they can take advantage of the higher availability of oxygen in air than in water. Bimodal species move from aquatic to aerial media and switch between habitats in response to environmental variations such as cyclical or anomalous temperature fluctuations. Here we tested the prediction that bimodal species cope better with thermal stress than truly aquatic species using the crab *Pachygrapsus marmoratus* as a model species. When in water, oxygen consumption rates of *P. marmoratus* acutely rise during warming. Beyond a temperature threshold of 23 °C the crab's aerobic metabolism in air remains lower than in water. In parallel, the haemolymph oxygen partial pressure of submerged animals progressive decreases during warming, while it remains low but constant during emersion. Our results demonstrate the ability of a bimodal breathing ectotherm to extend its thermal tolerance during air-breathing, suggesting that there are temperature-related physiological benefits during the evolution of the bimodal life style.

In ectothermic organisms, the capacity to extract oxygen from the environment and to deliver it effectively to tissues is a main determinant of the whole organism’s thermal tolerance^1^,^2^,^3^. Ectotherms can endure either abrupt or prolonged thermal variations if the integrated response of their respiratory and circulatory systems is able to cover the increase in oxygen demand during warming^4^,^5^,^6^. In contrast, when this response reaches its capacity, a mismatch between oxygen requirement and supply occurs and thermal tolerance is then constrained by the onset of progressive hypoxemia. These mechanisms constitute the core of the oxygen and capacity limitation of thermal tolerance (OCLTT) principle, a unifying concept that has found validation in many aquatic and some terrestrial ectotherms^1^,^2^,^3^,^4^,^7^,^8^,^9^,^10^ and has been able to explain phenomena observed in the field. Interestingly, the OCLTT theory predicts that the optimisation of respiratory mechanisms and increased oxygen availability would bring with it a widening of the thermal tolerance window. Experimental work on the Antarctic eelpout *Pachycara brachycephalum* has shown that exposure to hyperoxic water alleviates the fish’s heat-induced increase in cardiocirculatory cost and likely the associated loss of whole animal performance, moving the limiting threshold (the pejus limit) to higher temperatures than in individuals tested under normoxia conditions^11^. The optimisation of respiratory mechanisms and reduction of associated costs may thus play a crucial role in strategies adopted by eurythermal species when facing challenging fluctuations in environmental conditions, e.g., on an evolutionary scale when exploring new habitats^4^,^12^,^13^. For example, as demonstrated in the Green Crab *Carcinus maenas*, eurythermal decapods exploit haemocyanin-bound oxygen reserves during warming, compensating for the increased depletion of the dissolved oxygen fraction in the haemolymph and widening the window of aerobic scope across a wider range of environmental temperatures^4^.

On evolutionary time scales, the direct benefit of enhanced oxygen provision for improved thermal tolerance and increased efficiency of the delivery of oxygen to tissues suggests the emergence of novel respiratory modes^14^. As an example, increased heat tolerance is now regarded as a proximate cause for the recurrent and independent evolution of air-breathing species from water-breathing ancestors^15^. The emerging variety of respiratory modes has subsequently contributed to the diversification and complexity of life forms. Indeed, from an evolutionary point of view, the comparative evaluation of several aquatic insects has shown that increased respiratory control and the possibility of relying on aerial oxygen can mitigate the constraints on aerobic metabolism at higher temperatures and extend the realized thermal niche^16^,^17^.

These lines of evidence, however, leave open the questions of how and whether organisms with different respiratory modes exploit the benefits of both aquatic and aerial media and use both aquatic and aerial breathing depending on their habitat choice. Bimodal animals (also called dual breathers) are able to exchange oxygen and carbon-dioxide in both air and water. Aerial respiration in water-breathing forms evolved in response to factors in their aquatic habitats, such as low oxygen, high carbon dioxide and ammonia concentration as well as high temperatures, offering advantages especially in highly variable environments. In parallel, biotic factors including competition and predatory cues may stimulate animals to exploit terrestrial resources^18^. To date, only a few studies have considered warm temperatures as a driver for the emersion of aquatic animals^15^,^19^,^20^,^21^. In numerous taxa, modifications in respiratory apparatuses together with the evolution of novel structures are associated with the recurrent and convergent evolution of bimodal breathing^22^,^23^,^24^. Among vertebrates, nearly 400 species of bony fishes are able to switch to air-breathing, relying to various degrees on oxygen from both media^25^,^26^. A wide variety of non-vertebrate taxa show facultative bimodal breathing and these examples of adaptation are often regarded as stepping stones in the evolutionary transition from water to land^24^,^27^,^28^. Bimodal breathing evolved independently in several crustacean families and many such species populate the fresh- and sea-water/land boundary of all tropical and temperate regions^29^. Among them, decapods display the richest and most diverse radiation of dual breathing forms.

Against this background, the aims of the present study are: 1) to test the principle of OCLTT in an intertidal dual breather model species; 2) to provide experimental evidence for the benefits associated with retaining a bimodal life style at the air-water interface; and 3) to determine whether the environmental temperature leads the crabs to switch between different respiratory modes. We therefore utilised a data series on field temperature. Furthermore, we analyzed metabolic rates and oxygen partial pressures in the haemolymph under progressive warming conditions in animals breathing air or water. As a consequence, we were able to summarize in a conceptual model the benefits of the bimodal life style.

## Results

### Environmental temperature

**Figure 1:**
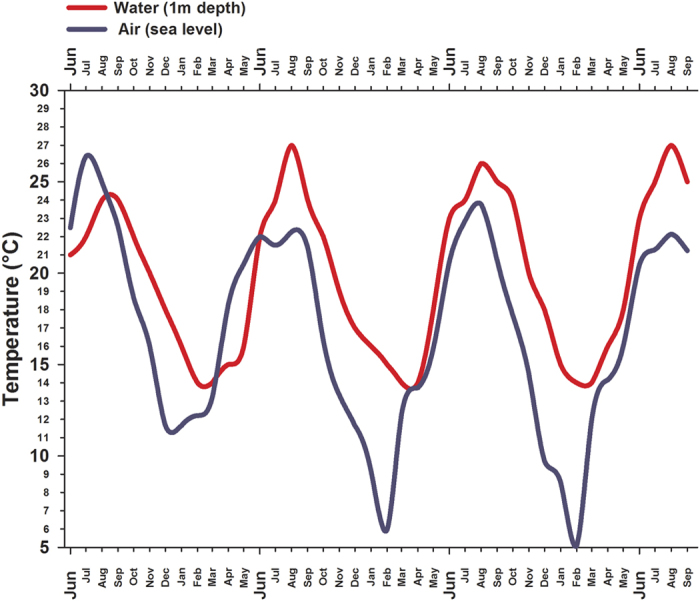
Field environmental temperature. Air- and Sea-Surface Temperature recorded at Calafuria site (Thyrrenian coast, Tuscany, Italy) from June 1997 to September 2000.

Field temperatures were characterized by strong seasonality and by mean water temperatures generally higher than mean aerial ones (Fig. 1). In particular, the ends of the summers were characterized by protracted warm conditions in the water compared to the aerial environment. Furthermore, the air temperature showed a greater variability than the water temperature.

### Oxygen consumption (MO_2_)

**Figure 2:**
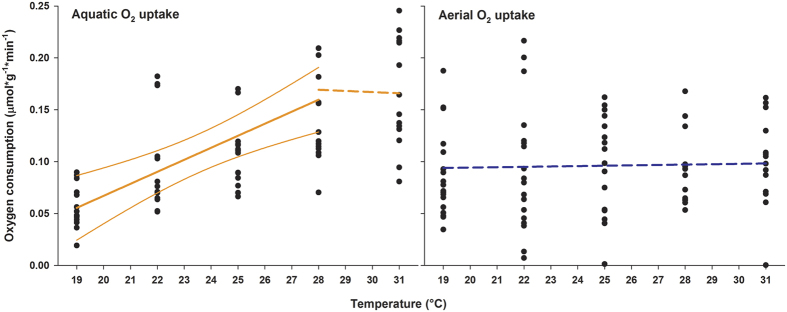
Oxygen consumption (MO_2_) rate of *Pachygrapsus marmoratus* in air and in water during acute warming. During aquatic oxygen uptake, warming caused a progressive rise in MO_2_ until capacity limits were reached at the critical threshold of 28 °C. In contrast, during aerial oxygen uptake, MO_2_ remained unchanged across the entire thermal ramp. Significant linear regression and the 95% confidence interval are represented by continuous, thick and normal lines, respectively. Non-significant linear regressions are represented by dashed lines. At each temperature 21 replicates were used (overlap may hide data points).

The oxygen consumption (MO_2_) of *Pachygrapsus marmoratus* in water significantly rose (linear model; df = 1,54; F = 37.49; p < 0.0001) up to 28 °C at which point piecewise two-segment linear regression found a significant discontinuity in the relationship. Above 28 °C (Fig. 2), the rise in MO_2_ levelled off, indicating that capacity and associated temperature limits were reached (linear model; df = 1,26; F = 0.11; p = 0.7426). In contrast, MO_2_ in air remained unchanged during acute warming (linear model; df = 1,81; F = 0.025; p = 0.875).

### Haemolymph oxygen partial pressure (PO_2_)

**Figure 3:**
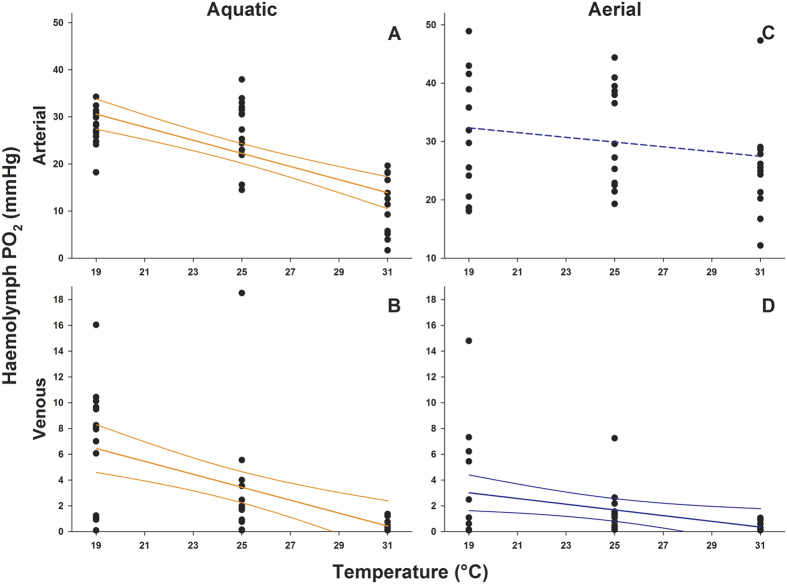
Temperature-dependent patterns of arterial and venous oxygen partial pressure in *Pachygrapsus marmoratus*. An acute increase of temperature induced a progressive decrease in the oxygen content in the haemolymph in all groups (**A,B** and **D**) with the exception of the arterial blood in crabs breathing air (**C**). In air, the possibility to sustain oxygen demand through efficient extraction from the environment prevents the onset of tissue hypoxemia and extends the thermal tolerance range. Significant regressions and the 95% confidence intervals are represented by continuous, thick and normal lines, respectively. The non-significant linear regression is represented with a dashed line. At each temperature 14 replicates were used (overlap may hide data points).

Acute warming caused a significant decrease in oxygen partial pressure in both the arterial and venous haemolymph when the crabs were breathing in water (Fig. 3A; GAM -General Additive Model, see methods section-; F = 19.91; p = 0.0002; Fig. 3B; GAM; F = 36.44; p < 0.0001;) and in the venous haemolymph when the animals were breathing in air (Fig. 3D; GAM; F = 6.08; p = 0.019). In contrast, when the crabs were breathing in air, their arterial oxygen partial pressure remained unchanged along the entire thermal ramp, indicating that oxygen provision was effectively sustained when oxygen uptake from the venous haemolymph was increased (Fig. 3C; GAM; F = 1.858; p = 0.169).

## Discussion

Taking environmental and physiological data together, our findings indicate that the bimodal species studied *Pachygrapsus marmoratus* can switch between different respiratory modes and shift its heat tolerance limits to higher temperatures by alternating between terrestrial and aquatic habitats. In aquatic environments, oxygen availability in relation to the capacity of the organism for oxygen uptake and use can become limiting during acute warming. The metabolism of *Pachygrapsus marmoratus* in water is largely temperature dependent, as revealed by the increase in oxygen consumption (MO_2_) between 19° and 28 °C. Thus, warmer water temperatures beyond the pejus limit cause a decline in aerobic scope, due to the increased cost of oxygen supply and associated oxygen demands until capacity and critical limits are reached. The pejus limit sets in before the crab’s anaerobic metabolism becomes involved beyond the critical temperature of 28 °C, when its respiration rate begins to decline, which is compensated by the animal’s anaerobic metabolism and possibly by its associated metabolic depression. From estimated temperature coefficients, the thermal response of MO_2_ in *P. marmoratus* (Q_10[19°–28°C]_ = 3.4) is comparable to that in other marine eurytherms such as *Sepia officinalis* (Q_10[8°–23°C]_ = 2.8)^9^, *Zoarces viviparous* (Q_10[6°–24°C]_ = 3.1)^11^, and *Arenicola marina* (Q_10[9°–24°C]_ = 2.1)^1^. The oxygen partial pressure (PO_2_) in the haemolymph is in line with the onset of hypoxemia at high temperatures. Similar to other marine crustaceans, *P. marmoratus* shows a marked decrease in blood oxygenation at the critical thermal threshold even on the arterial side^8^,^30^. Thus, in agreement with available data for other marine organisms, the thermal niche of *P. marmoratus* in water appears primarily constrained by the early mismatch between oxygen uptake and demand during acute warming below critical temperatures.

In contrast, when acute warming occurs in air, the crabs exhibit a totally different thermal response. Their oxygen uptake and arterial haemolymph PO_2_ remain stable over the entire temperature range. In particular, the patterns of blood oxygen tension demonstrate that *P. marmoratus*, similar to terrestrial arthropods, does not experience obvious increments in its resting metabolic rate during acute warming. Similar effects were seen in fish, bivalves, gastropods and aquatic insects exposed to hyperoxia during warming^11^,^31^,^32^,^33^. Thus, the higher availability and diffusiveness of oxygen in air allows the animal to cover demand at lower cost when temperature rises. Overall, our experiments support the general principle of OCLTT for bimodal species such as *P. marmoratus* and also demonstrate that their oxygen supply capacity covers their resting oxygen demand more easily while breathing air. This suggests a widening of the thermal tolerance window in air under resting conditions and, particularly, the sustenance of the animal’s aerobic scope at higher temperatures. Our results demonstrate, in fact, that the bimodal species *P. marmoratus* is able to exploit two different thermal niches. While ectotherms fully breathing either air or water are confined to a single respiratory medium, the possibility of switching between gaseous and dissolved oxygen confers a striking metabolic advantage to the bimodal species *P. marmoratus*, especially in habitats such as intertidal zones, where solar heating is one of the most important driver of drastic temperatures fluctuations.

**Figure 4:**
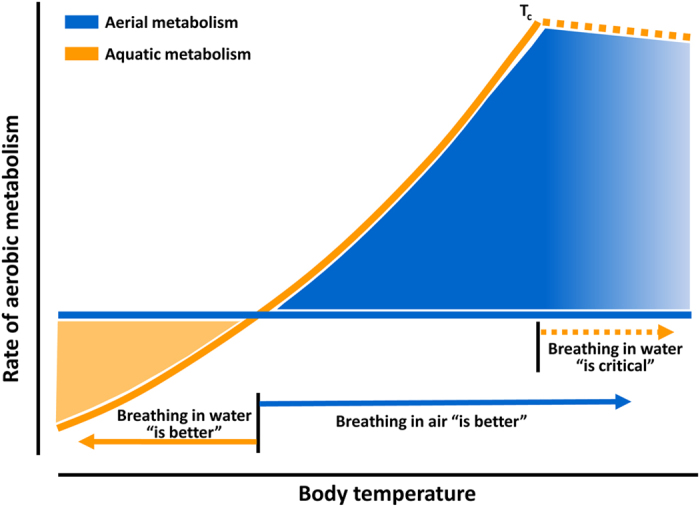
Conceptual model of the temperature-dependent aerobic metabolism in bimodal animals. The rate of aerobic metabolism in water rises with temperature (orange line). In contrast, aerobic metabolism in air remains steady in the same body temperature range (blue line), indicating that in bimodal species, the oxygen and capacity limits of thermal tolerance shift to warmer temperatures in air. The strategy to minimize energy expenditure in bimodal species differs along the thermal gradient. On the left side of the intersection point of the metabolic rate lines, elevated oxygen uptake in air is determined by the energetic cost required to sustain body posture during aerial exposure (orange area, “Breathing in water is better”). On the right side, MO_2_ acutely rises to sustain aerobic metabolism in water and exceeds that in air (blue area, “Breathing in air is better”). Further warming beyond the critical temperature (T_c_) generates the onset of hypoxemia in submerged animals, due to progressive failure of oxygen provision (“Breathing in water is critical”). The area on the right side of T_c_ is represented faded because the exact distinction between aerial and aquatic metabolism is no longer possible due to the progressive rise of anaerobic metabolism. This model has practical applications and explains the bimodal life style in animals that live at the air-water interface.

These results are suggestive of temperature-related benefits from colonising terrestrial habitats enabled by maintaining a bimodal respiration capability. This study leads to several consecutive hypotheses. In the proposed conceptual model in Fig. 4, below a body-temperature threshold represented by the intersection point between aerial and aquatic MO_2_, *P. marmoratus* maintains a lower metabolic rate in water than in air, suggestive of better performance in water (indicated as “breathing in water is better”). As a benefit, activity under water is supported by enhanced buoyancy, which counterbalances the effect of gravity and reduces the operative weight of submerged grapsid crabs to one-tenth of their land weight^34^,^35^. This underwater micro-gravity condition would come with lower energy expenditure required to sustain body posture as suggested by the high value of venous oxygen content (Fig. 3B). Warming water, however, increases the animal’s aquatic metabolic rate, which then rapidly exceeds its metabolic rate in air. Thus, at body temperatures higher than the intersection point of these two metabolic trajectories, the animal’s aerial metabolism is more efficient in maintaining aerobic performance and providing thermal tolerance (indicated as “breathing in air is better” in Fig. 4). The further warming beyond critical temperature, exacerbates the hypoxemic condition of water-breathing animal determining the onset of anaerobic metabolism (indicated as “breathing in water is critical” in Fig. 4).

The intertidal habitat is characterized by remarkable spatial and temporal variations in air and water temperatures that determine complex mosaics of microclimates and that shape life traits and strategies of coastal animals^36^,^37^. Our findings provide strong support to the hypothesis that the bimodal life strategy of organisms that move through emerged or submerged areas represents an adaptation to efficiently face the thermal heterogeneity of such environments. Specifically, for animals acclimated at 19 °C, the comparison of field data with the 23 °C threshold, i.e. the intersection point between aerial and aquatic metabolism (Fig. 2), is suggestive of the seasonal habitat selection by *P. marmoratus* (Fig. 4). Indeed, sea water temperatures higher than 23 °C occur during the summer months and often prevail until autumn. This would induce crabs to leave the water to alleviate thermal stress while breathing air. It is worth mentioning that this temperature not only represents the upper margin for the animal’s energy-saving metabolism in water but, in this species, it also corresponds to the onset of the heat-shock response upon thermal stress^38^,^39^. Thus, the onset of hypoxemia at the pejus limit may trigger the activation of the heat shock response as observed in other invertebrates^1^,^2^,^3^,^40^. It should be specified that the environmental air temperature rarely matches the organism’s body temperature and that only the latter determines the metabolic rate in air. Intertidal organisms are subjected to several environmental influences, such as solar heating, that may increase the body temperature far above the air temperature^37^. However, bimodal mobile organisms not only facultatively move between mediums but are able to adopt a vast array of thermoregulatory mechanisms that efficiently prevent excessive heating. Intertidal crabs, for example, effectively dissipate heat trough their carapace^42^,^43^ or control their body temperature by actively moving to moist and shady microhabitats^44^. Further studies are required to validate these findings, testing the hypothesis of the evolution of bimodal life in response to thermal heterogeneity in an array of species of different taxonomic groups.

In summary, our findings explain the preference of *P. marmoratus* for aerial habitats during summer and early fall and for a submerged lifestyle during late autumn, winter and spring when water temperatures fall below the limiting values (Fig. 1). During the colder seasons, the aquatic lifestyle is supported by the thermally buffered aquatic environment (the temperature never falls below 14 °C); at the same time, there is no energetic benefit from being in air during the cold season when the air is colder than the water. Such cooling may impair animal performance and fitness on the cold side of the thermal window^2^,^8^,^45^. Conversely, during the warmer season, water temperatures rising above 23 °C can explain the semi-terrestrial behaviour adopted by *P. marmoratus* since the movement to an aerial environment would minimize its warming-induced rise of metabolic expenditure and thermal stress. It should be noted that the temperature threshold responsible for the bimodal strategy may shift depending on the animal’s acclimation temperature and that this temperature has to be accurately determined in different cases by taking into account the physiological capacities of the studied animals and the environmental features of the selected habitat. In addition, the model proposed for the seasonal transitions between different media of *P. marmoratus*, would narrowly apply for all other species that rely on bimodal life strategy on daily basis or in response to acute and anomalous climatic events. Future studies would address the role of seasonal acclimation temperatures in determining different thresholds of transition between aquatic and aerial media, investigating the influence of thermal conditions on the potential plasticity of the bimodal life strategy in various bio-geographic contexts.

## Perspective

Our conceptual model contributes to understanding the ecology and biology of bimodal species such as *P. marmoratus*. Spatial and temporal variations in the physical characteristics of the water body, the tidal regime, the type of substrata, solar heating and other abiotic factors are also expected to shape the selective use of aerial vs. aquatic media by dual breathers. The fauna of intertidal areas includes a variety of bimodal organisms. Their ability to exploit both the aquatic and aerial phases has to be taken into account in models and scenarios predicting the consequences of climate warming and local thermal variations on their present and future distribution and biology.

## Methods

*Pachygrapsus marmoratus* (Fabricius, 1787) (Brachyura, Grapsidae), one of the most abundant crabs in the intertidal and subtidal zones of the rocky shores of the Mediterranean and European Atlantic Oceans, has been selected as a dual-breather model species^38^,^41^. *P. marmoratus* has long been studied for its pivotal role in the ecology of rocky shore areas because of its diet and control over the community structure of other intertidal species^46^,^47^. Able to breathe in air and water, either when active or inactive, *P. marmoratus* exhibits a truly semi-terrestrial physiology^34^,^46^. In addition, this species presents a convincing case study for the bimodal lifestyle because it shows a seasonal pattern of habitat selection. Generally, it inhabits the upper tidal belt where it is fully exposed to air during the summer season, whereas during the colder seasons, it usually moves into tidal pools or below the subtidal fringe by switching to a fully aquatic lifestyle^34^,^46^,^48^. These observations are further supported by the foraging behaviour of *P. marmoratus*, which feeds mainly on subtidal algae during the winter and on intertidal life forms during the warm spring and summer months^47^,^49^.

Specimens of both sexes (the female were not gravid) and of the same sizes were hand-collected during the spring from the rocky shore of Calafuria (43°30′ N, 10°20″ E) in the Ligurian Sea, Italy, and immediately transported to the rearing facilities of the Department of Biology in the University of Florence, where they were kept in aquaria with filtered, aerated re-circulating natural seawater at 19 ± 0.2 °C, 38‰ salinity and a 12 h light/dark cycle for at least four weeks before the experiments began.

All experiments described in the manuscript were conducted in accordance with the Guidelines for the Treatment of Animals in Behavioral Research and Teaching from the Animal Behavior Society^50^. Animals used in our experiments were maintained and treated in compliance with the guidelines specified by the Italian Ministry of Forestry and Agriculture and Biology Department at the University of Florence. In addition, all necessary permits were in hand when the research was conducted and all experiments and procedures were approved by the University of Florence.

### Environmental temperature

Historical series of air temperatures were obtained from the Institute of Biometereology (IBIMET, CNR - Italy) that runs weather stations in the study area. Among the stations that monitor the Tuscany coast, the one located close to the Calafuria inlet profiles the temperature on the rocky shore from a height of 1 m above sea level. Water temperatures were retrieved from a study performed in the same area (43°30′ N, 10°20″ E) by Bramanti and collaborators^51^. The reported water temperatures are the onshore temperatures detected in the first meter of water depth recorded by the Tuscany Environmental Agency (ARPAT) station using a multiparametric Idronaut probe (Mod. Ocean Seven 316). Since these sources of air and water temperature series do not entirely overlap, only the overlapping range between June 1997 and September 2000 was considered in this study.

### Oxygen consumption rate (MO_2_)

Five custom-made Perspex chambers (0.5 litres) were submerged in water in large-volume holding tanks and were used to measure the routine MO_2_ of individual crabs in air and in water. The submersion of the respiratory chambers in a temperature-controlled water bath guaranteed that both aerial and aquatic MO_2_ measurements were performed under steady thermal regimes and that the crabs’ body temperature constantly match the temperature of the surrounding medium. During the crab’s aerial respiration, the chamber was supplied with humidified air whereas during the crab’s aquatic respiration, the chamber was supplied with water with greater than 95% oxygen saturation. The respiration chambers were darkened with aluminium foil to minimize the reactivity of the animals, and the chambers were immersed in a water bath to set the experimental temperatures. Fibre-optic oxygen meters (Fibox 3 with oxygen Microoptode Type PSt3 PreSens, Regensburg, Germany), calibrated in air-equilibrated seawater (100%) and a saturated sodium thiocyanate solution (0%), were connected to the external wall of the chambers by matching an internally glued oxygen sensor spot in accordance with the supplier’s instructions.

An intermittent flow between the chambers and the holding tank was regulated with circulation pumps and the MO_2_ was calculated from the decrease in oxygen content in the chambers over a given time during the stationary phase of the flux, ensuring that the oxygen concentration never fell below 60% saturation. During the aquatic respiration measurements, the movements of the crabs were adequate to maintain water mixing inside the chambers, allowing linear recordings of the oxygen decline over time. An additional chamber (control) filled with air or water was used to quantify the background MO_2_, which was routinely less than 2–3% of the consumption recorded in water, and negligible in air. The crabs were starved for at least 24 h prior to their introduction into the chambers and allowed to settle for an additional 6 h to reduce possible disturbance effects such as specific dynamic action or handling stress. Prior to the experiments, individual crabs were placed in the chambers and allowed to recover overnight from handling stress. Subsequently, their MO_2_ was determined at 19 °C/22 °C/25 °C/28 °C/31 °C in a stepwise procedure along a 1 °C × h^–1^ temperature ramp. Following each experiment, the animal wet weight was measured and the animal volume was calculated by immersing individuals into a graduated measuring cylinder to measure the volume of displaced water.

### Haemolymph oxygen partial pressure

Measurements of arterial and venous PO_2_ were performed with fiber-optic oxygen microsensors (NTH-PSt1-L5lTF-PC3,1-NS 35x1,20-YOP, PreSens GmbH, 93053 Regensburg, Germany) and recorded online with a TX2-A oxygen meter (PreSens) with integrated signal processing software. Prior to each experiment, the optodes were calibrated in oxygen-free (addition of sodium dithionite) and air-saturated (bubbled) seawater. The animals were starved for 72 h and acclimated for 16–24 h in the experimental tank prior to measurements of dissolved oxygen at 19 °C/25 °C/31 °C. The temperature was increased as described above. All experiments and the thermal ramp were performed in a temperature-controlled room in which the environmental temperature was maintained with an accuracy of ± 1 °C.

Small haemolymph samples (less than 20 μl) were drawn by capillary action into a manually sharpened Pasteur pipette in which the oxygen sensor was previously inserted and positioned close to the tip. To measure the oxygen partial pressure in post-branchial haemolymph (arterial), the tip of the pipette was inserted through a hole (maximum width 0.2 mm) drilled in the carapace over the pericardial sinus, avoiding injury to the hypodermis, according to Giomi and Pörtner^4^. Venous blood was withdrawn from the sinus below the arthrodial membrane at the base of the fourth or fifth pereiopod.

### Statistical analysis

To describe temperature-dependent trends in MO_2_ patterns and haemolymph oxygen content, linear regressions were utilised throughout. In addition, to verify possible changes in MO_2_ trends, a two-parameter piecewise function was implemented. All these regression fits were performed with the SigmaPlot 11.0 package. The temperature coefficient (Q_10_) adopted to express the variation in MO_2_ between two different temperatures was calculated using the equation Q_10_ = (Rate_T2_/Rate_T1_)^(T2–T1)/10^. To test for statistically significant relationships between air/water temperatures and MO_2_, we tested 21 specimens in each treatment, and a linear model was used that considered MO_2_ as the response variable and temperature as the continuous explanatory variable. To normalize MO_2_, a square root transformation was performed and the normality was tested using the Shapiro-Wilk test (W = 0.988, p = 0.24). To test the statistical significance of PO_2_ in air and water in both arterial and venous haemolymph, we tested 14 specimens in each treatment. Since the data were not normal, a non-parametric technique, the Generalized Additive Model (GAM), was used by considering PO_2_ as the response variable and temperature as the continuous explanatory variable. All statistical analyses were carried out using R^52^.

## Additional Information

**How to cite this article**: Fusi, M. *et al*. The trade-off between heat tolerance and metabolic cost drives the bimodal life strategy at the air-water interface. *Sci. Rep.* **6**, 19158; doi: 10.1038/srep19158 (2016).
